# Fluorescence-Based High-Throughput Functional Profiling of Ligand-Gated Ion Channels at the Level of Single Cells

**DOI:** 10.1371/journal.pone.0058479

**Published:** 2013-03-08

**Authors:** Sahil Talwar, Joseph W. Lynch, Daniel F. Gilbert

**Affiliations:** 1 Queensland Brain Institute, The University of Queensland, Brisbane, Queensland, Australia; 2 School of Biomedical Sciences, The University of Queensland, Brisbane, Queensland, Australia; 3 Institute of Medical Biotechnology, Friedrich-Alexander-University Erlangen-Nuremberg, Erlangen, Germany; Sackler Medical School, Tel Aviv University, Israel

## Abstract

Ion channels are involved in many physiological processes and are attractive targets for therapeutic intervention. Their functional properties vary according to their subunit composition, which in turn varies in a developmental and tissue-specific manner and as a consequence of pathophysiological events. Understanding this diversity requires functional analysis of ion channel properties in large numbers of individual cells. Functional characterisation of ligand-gated channels involves quantitating agonist and drug dose-response relationships using electrophysiological or fluorescence-based techniques. Electrophysiology is limited by low throughput and high-throughput fluorescence-based functional evaluation generally does not enable the characterization of the functional properties of each individual cell. Here we describe a fluorescence-based assay that characterizes functional channel properties at single cell resolution in high throughput mode. It is based on progressive receptor activation and iterative fluorescence imaging and delivers >100 dose-responses in a single well of a 384-well plate, using α1-3 homomeric and αβ heteromeric glycine receptor (GlyR) chloride channels as a model system. We applied this assay with transiently transfected HEK293 cells co-expressing halide-sensitive yellow fluorescent protein and different GlyR subunit combinations. Glycine EC_50_ values of different GlyR isoforms were highly correlated with published electrophysiological data and confirm previously reported pharmacological profiles for the GlyR inhibitors, picrotoxin, strychnine and lindane. We show that inter and intra well variability is low and that clustering of functional phenotypes permits identification of drugs with subunit-specific pharmacological profiles. As this method dramatically improves the efficiency with which ion channel populations can be characterized in the context of cellular heterogeneity, it should facilitate systems-level analysis of ion channel properties in health and disease and the discovery of therapeutics to reverse pathological alterations.

## Introduction

Ion channels are involved in most physiological and disease processes [1]–3 and are considered highly attractive drug targets for therapeutic intervention [4]–[7]. Their biophysical and pharmacological properties are determined by the combination of subunits, which in the case of ligand-gated channels is often heterogeneous. It can change during development, in a tissue-specific manner or as a consequence of pathophysiological events [8]–[12]. It is necessary to characterise the properties of ion channels in the context of cellular heterogeneity as a prerequisite to understanding their physiological and pathological roles, and to understand the effects of drugs on them [13], [14].

The biophysical and pharmacological properties of ligand-gated ion channels are typically evaluated by analysing activation and inhibition concentration-responses and derived measures, in particular half-maximal activation or inhibition concentration (EC_50_, IC_50_), hill coefficient (nH or slope) and dynamic range of the response. While there are various technologies available that allow concentration-response experimentation with ion channels, such as flow cytometry [15], [16], dynamic mass redistribution [17] or radioactive, non-radioactive and spectroscopic measurements the most commonly applied methods are patch clamp electrophysiology and fluorescence-based functional imaging [7], [18]–[20].

Patch-clamp electrophysiology is referred to as the gold standard of ion channel evaluation as it yields data of unsurpassed spatiotemporal resolution and allows analysis at the level of single cells and even single channels [21], [22]. Despite advances in the field of high-throughput electrophysiology, patch-clamp electrophysiology remains labour intensive, requires highly skilled staff and only supports a small number of individual experiments, hence is not readily applicable to large-scale systematic approaches with heterogeneous cell samples and chemical or genetic libraries [5], [23].

Fluorescence-based evaluation of ion channels in live cell assays is typically conducted on hundreds to thousands of individual cells using multimode fluorescence reader of high sensitivity that are fast but low in spatial resolution. This method, usually implemented and scaled to high-throughput format, yields robust signals and enables establishment of screening assays with large dynamic range [24] but is limited with regard to single cell-resolution, due to the stochastic average being masked by bulk measurements [25]. Consequently, this technology is inappropriate for functional screening analysis of ion channels in the context of cellular heterogeneity.

To bridge the gap between the limited throughput of patch-clamp electrophysiology and the low resolution of conventional fluorescence-based high-throughput screening techniques, we aimed to establish a method that allows large-scale functional analysis of ion channels at the level of single cells and in high-throughput mode. To this end, we aimed to adapt a YFP_I152L_-based protocol for functional analysis of glycine receptor chloride channels (GlyRs) in recombinantly modified HEK293 cells.

YFP_I152L_, an engineered variant of YFP with greatly enhanced anion sensitivity, is quenched by small anions and is thus suited to reporting anionic influx into cells [26]. It has proved useful in screening compounds against many chloride channel types [18], [24], [27].

The GlyR, which is a member of the pentameric Cys-loop ion channel receptor family, mediates inhibitory neurotransmission in the central nervous system. Functional GlyRs are formed from a total of five subunits (α1–α4, β) which assemble either as α homomeric or αβ heteromeric channels. Biochemical, biophysical, pharmacological and genetic evidence suggest the majority of glycinergic neurotransmission in adults is mediated by heteromeric α1β GlyRs although homomeric GlyRs are also expressed in different tissues at varying abundance [10]. However, αhomomeric GlyRs might be more prevalent than previously assumed given that β subunit expression has recently been shown to be low in the mouse neocortex, cerebellum and hippocampus [28].

Although GlyRs are not yet targeted by any clinically useful drugs, they have recently emerged as potential therapeutic candidates for neurological disorders including epilepsy [29]–[31], peripheral inflammatory pain [32], spasticity and hyperekplexia [33]. New drugs that specifically modulate (i.e., inhibit or potentiate) different GlyR isoforms may thus be useful as both therapeutic lead compounds and as pharmacological probes for basic research. In addition GlyRs and other members of the Cys-loop ion channel receptor family have been implicated as neurotoxicity targets for solvents [34] and insecticides [35] and are increasingly acknowledged in the context of cancer [3], [36].

In this article, we describe a fluorometric technique that allows automated large-scale concentration-response experimentation with GlyRs in the context of cellular heterogeneity to address the issues encountered in the classical approaches described above. We assessed the quality of the assay by correlating acquired EC_50_ values to functional characteristics obtained from patch-clamp electrophysiology and evaluated its suitability for operation in high-throughput mode. We further aimed to conduct a case study with the insecticide lindane in pure and mixed cell populations to assess the usability of the employed assaying technique for clustering GlyR phenotype populations and for identification of subunit-specific drugs in heterogeneous cell cultures.

## Materials and Methods

### Cell Culture

Experiments were performed on HEK293 cells cultured in Dulbecco’s modified Eagle’s medium (DMEM) supplemented with 10% fetal calf serum and 1% penicillin/streptomycin. Approximately 5×10^5^ cells suspended in 5 mL DMEM were.

plated into 60-mm culture dishes and were transfected when approx. 80% confluent. YFP and GlyR chloride channel subunits were transfected in 1∶1 (YFP and α1-3 GlyR homomers) or 2∶1∶1 ratio (YFP, GlyR α1-3 homo and α1-3β GlyR heteromers) with a total cDNA quantity of 1 µg. Cells were transfected using calcium phosphate precipitation transfection technique, as described in Sambrook et al. [37]. Transfection efficiency varies from 20 to 60%. We routinely achieve a correlation of 80 to 90% between cells expressing YFP and cells expressing GlyRs. We have published a detailed comparison of five transient transfection methods employed routinely in the laboratory for this purpose [27].

### Molecular Constructs

The human α1, α2 and α3 GlyR plasmid DNAs were subcloned into the pcDNA3.1 plasmid vector and the human β subunit plasmid DNA was subcloned into pIRES2-EGFP plasmid vector. The UniprotKB accession numbers for the DNAs are as follows (with gene name in brackets): α1 (GLRA1): P23415, α2 (GLRA2): P23416, α3 (GLRA3): O75311 and β(GLRB): P48167.

### Cell Lines

Hek293 cells (CRL-1573™) were purchased from *The American Type Culture Collection* (ATCC).

### Preparation of Cells for Experiments

Following termination of transfection, cells were trypsinized by adding 0.7 mL of 0.25% trypsin–EDTA solution (Gibco BRL), resuspended into DMEM, and 2.5×10^3^ cells, suspended in 40 µL DMEM, were plated into each well of a transparent 384-well plate for fluorescence imaging experiments. Cells were used in experiments 24 h later. Individual wells typically contained 5×10^3^ cells at the time of the experiment. Approximately 1 h prior to commencement of experiments culture media in 384-well plates were removed by turning the plate upside-down onto a stack of tissue and were left for approximately 30 sec until culture media was entirely removed from the wells. The culture media was replaced by 20 µL standard control solution, which contained (in mM) NaCl 140, KCl 5, CaCl_2_ 2, MgCl_2_ 1, HEPES 10, and glucose 10 (pH 7.4) using NaOH. The NaI test solution was similar in composition to NaCl control solution except the NaCl was replaced by equimolar NaI. For repeated receptor stimulation with increasing agonist concentration NaI test solution was supplemented with 30 mM glycine and serially diluted with NaI test solution to obtain agonist solutions containing 0.1, 0.3, 1, 3, 10, 30, 100, 300, 1000, 3000, 10000 and 30000 µM final glycine concentration. For drug screening experiments agonist solutions were supplemented with strychnine (10, 30, 100 µM), picrotoxin (10, 100 µM) and lindane (10, 100 µM) respectively. Drugs were diluted from stocks in agonist solution according to desired final concentrations. The final concentration of applied drugs was adjusted for high-throughput drug screening that is typically conducted at concentrations in the micromolar range [38], [39]. Experiments were conducted at room temperature (19–22°C).

### Pharmacological Reagents

Glycine, picrotoxin, lindane and strychnine were obtained from Sigma-Aldrich Chemie GmbH (Steinheim, Germany). Picrotoxin and strychnine were prepared as 100 mM stocks in dimethylsulphoxide (DMSO). Lindane was prepared as 30 mM stock in DMSO and glycine was prepared as a 1 M stock in water. All stocks were frozen at −20°C. From these stocks, solutions for experiments were prepared on the day of recording.

### Imaging Infrastructure

For imaging experiments we have designed an automated fluorescence microscope (Olympus IX51 inverted microscope) equipped with a motorized stage (Prior ProScan II, Prior Scientific Instruments, Cambridge, UK), a CCD camera (CoolSNAP monochrome cf/OL, Olympus) an LC PAL autosampler (CTC Analytics, Zwingen, Switzerland) with 100-µL syringe and a 100-W mercury arc lamp (HBO 103/2, Osram, Germany) for illumination. A suite of LabView 2010 (National Instruments, Ireland) software routines purpose written was used for hardware control, image acquisition, data storage and image analysis [24].

### Imaging Experiments

The 384-well plates were placed onto the motorized stage of our in house-built imaging system and cells were imaged with a 10× objective (UPlanFLN, N.A. 0.30, Olympus,Tokyo, Japan). Illumination from a mercury arc lamp, passing through a YFP dichroic mirror (86002V2 JP4 C76444, Olympus), was used to excite YFP fluorescence. Fluorescence was imaged by a CCD and digitized to disk onto a personal computer. The primary resolution of the camera was 1392×1040 pixels, although images were binned (2×2), resulting in a resolution of 696×520 pixels.

The maximum image acquisition rate was 1.25 Hz. Solutions containing agonist and drug were pipetted into a 384-well plate and placed onto LC PAL sample holder platform. Liquid handling was performed with the autosampler described above. Solutions were applied to cells at a rate of 0.6 ml/min. The experimental protocol involved imaging each well eleven times: once in 20 µl control solution and ten times 5 sec after the injection of 10 µl NaI agonist solution containing increasing concentrations of glycine and, when used, constant drug concentrations.

### Image Analysis

Images of fluorescent cells were segmented and quantitatively analyzed using a modified version of DetecTIFF® software [40]. In brief, images were segmented using an iterative size and intensity-based thresholding algorithm and the fluorescence signal of identified cells was calculated as the mean of all pixel values within the area of a cell.

### Calculation of Concentration-response Relationships

Individual concentration responses were constructed from image series of the same cells exposed to increasing glycine concentrations, resulting in 11-point dose responses for each of the imaged cells. As only those cells near the centre of the well were evaluated each image typically contained 100 to 500 fluorescent cells revealing the same number of agonist concentration responses for each tested well of a 384-well plate. Recorded single cell-derived glycine concentration-response relationships were fitted with the LabView VI *nonlinear curve fit.vi*. This VI uses the Levenberg-Marquardt algorithm to determine the set of parameters that best fit the set of input data points (X, Y) as expressed by the nonlinear function:
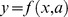
where *a* is the set of parameters. The curve model is defined by the following equation:



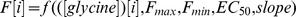
where F is the fluorescence value corresponding to a particular glycine concentration, [glycine]; F_min_ and F_max_ are the fluorescence values at highest and lowest agonist concentration, respectively; EC_50_ is the concentration that elicits half-maximal activation; and slope is the incline of the sigmoidal curve at half-maximal activation.

### Data Filtering

To exclude data recorded from biologically irrelevant objects calculated concentration response characteristics were filtered by the following parameters:

1. Goodness of fit (R^2^, ≥0.9), to include curve fits that represent the data points to a high degree.
2. Dynamic range of fluorescence change (ΔF, 20–100%), to include cells co-expressing both, YFP_I152L_ and GlyRs. The dynamic range (% fluorescence change) is defined as

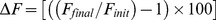
where F_min_ and F_max_ are the fluorescence values at highest and lowest agonist concentration, respectively.
3. Slope of the curve at half-maximal receptor activation (slope, 0.5–5), to include a wide range of physiologically relevant values.
4. Half-maximal concentration of receptor activation (EC_50_, 0.3–3000 µM), to allow for a large range of experiments with different cell lines and drugs shifting the EC_50_ towards higher agonist concentrations.

Filter ranges for enrichment of functional data were determined empirically by biological relevance. If not stated otherwise, averaged EC_50_ values are displayed as median +/− SEM.

### Clustering of Functional GlyR Phenotypes in Heterogeneous Cell Cultures

Functional parameters (EC_50_, slope) of training and test sets were annotated in Microsoft Excel. The model for phenotype classification was computed by a 10-fold cross-validation of the training set using J48 decision tree algorithm [41] available in WEKA 3.6 software [42]. Predicted phenotypes were saved in *.csv format and displayed with Origin 7G.

### Whole-cell Patch-clamp Electrophysiology

Cells were transfected as described above and plated onto glass coverslips in 3 cm culture dishes. Coverslips containing cells were placed in a recording chamber on the stage of an inverted fluorescent microscope and currents were recorded using the whole-cell patch-clamp configuration. Cells were perfused by extracellular solution containing (in mM): 140 NaCl, 5 KCl, 2 CaCl_2_, 1 MgCl_2_, 10 HEPES/NaOH and 10 glucose (pH 7.4 adjusted with NaOH). Patch pipettes were fabricated from borosilicate hematocrit tubing (Hirschmann Laborgeraete, Eberstadt, Germany) and heat polished. Pipettes had a tip resistance of 1–2 MΩ when filled with the intracellular solution consisting of (mM): 145 CsCl, 2 CaCl_2_, 2 MgCl_2_, 10 HEPES and 10 EGTA (pH 7.4 adjusted with CsOH). After establishment of the whole-cell recording configuration, cells were voltage clamped at −40 mV and membrane currents were recorded using an Axopatch 1D and pClamp 10 software (Molecular Devices). Currents were filtered at 500 Hz and digitized at 2 KHz.

Because αsubunits can form functional receptors with or without β subunits, it was necessary to confirm that the expressed receptors incorporated β subunits. As the GlyR β subunit cDNA was cloned into the pIRES2-EGFP plasmid vector, we used green fluorescent protein fluorescence to identify cells transfected with the GlyR β subunit. The successful incorporation of β subunits into functional heteromeric GlyRs was also confirmed pharmacologically by their characteristic reduction in lindane sensitivity as described below.

### Statistical Analysis

The statistical significance between two independent experimental groups was determined by unpaired Student’s t-test, with p<0.05 representing significance.

## Results

**Figure 1:**
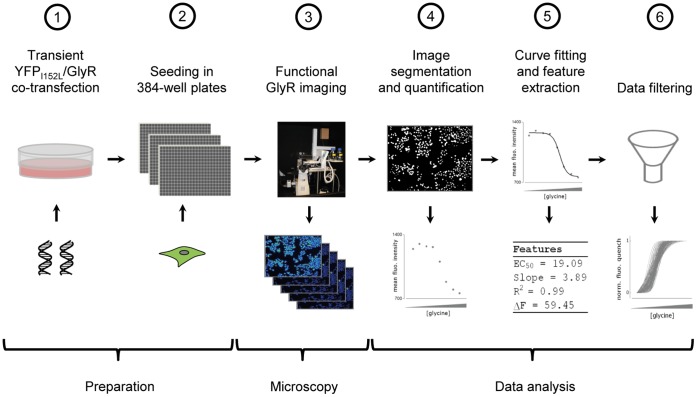
Work flow of experiment and data analysis. HEK293 cells were transiently co-transfected with YFP_I152L_ and GlyR cDNA (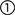). Approximately 48 h later, cells were seeded into the wells of 384-well plates at defined density and are cultured for another 24 h (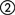). Functional analysis of GlyRs is carried out by progressive receptor activation and iterative fluorescence imaging using an in house-built automated screening device with integrated liquid-handling robotics (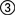). Recorded images are segmented and fluorescence dose-responses calculated (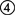) are fitted (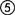). Finally, functional parameters measured in single cells, such as R^2^, ΔF, slope and EC_50_ are filtered to discriminate functional from non-functional data (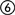).

We have established a fluorometric, YFP_I152L_-based screening assay for massively parallel functional analysis of GlyRs in individual cells, providing >100 glycine dose-responses in a single experiment. The assaying approach is based on progressive receptor activation by application of increasing agonist concentration followed by imaging and quantitative analysis of cellular YFP signal, including image segmentation and quantification, curve fitting and rigorous filtering of parameters derived from dose-responses to discriminate unreliable from physiologically plausible data. The experimental workflow is shown in Fig. 1.

### Assay Development

**Figure 2:**
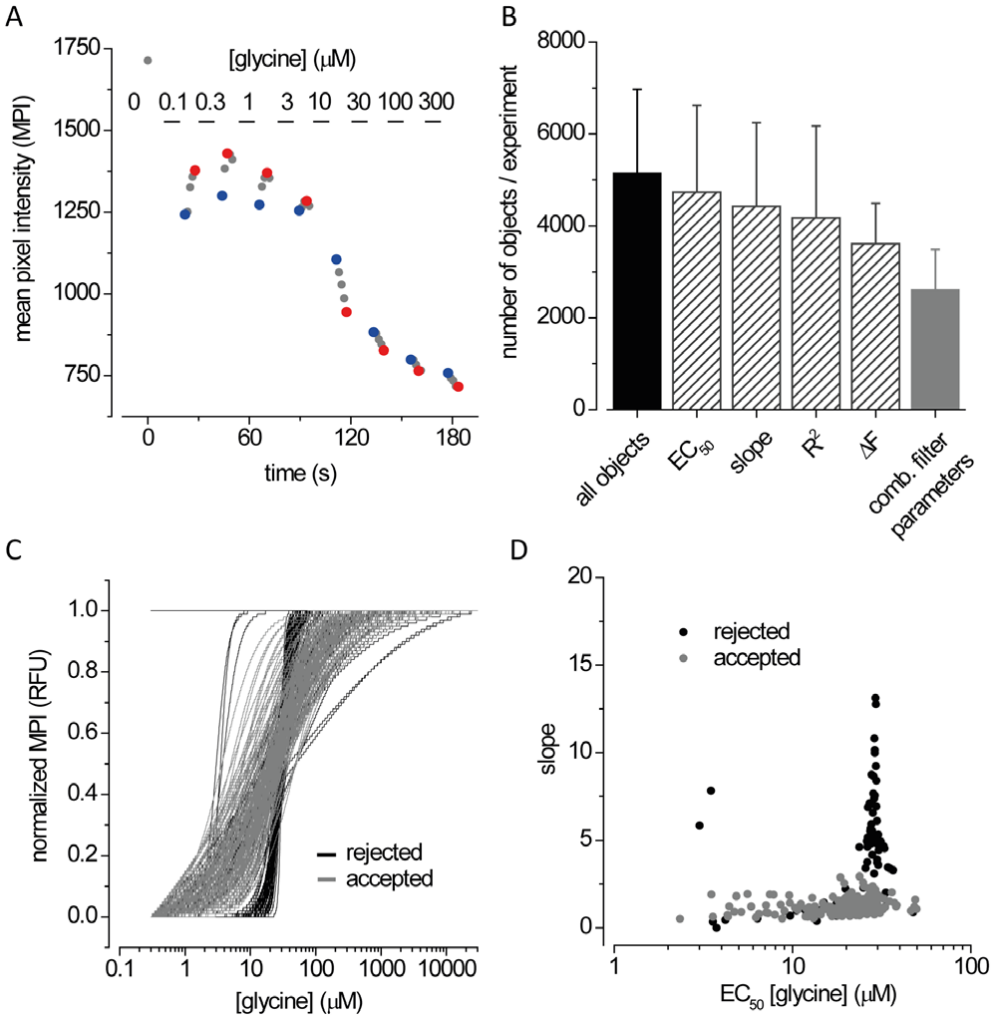
Development of the assay for single cell-based functional imaging. **A.** Determination of optimal assay conditions. HEK293 cells YFP_I152L_ and α1 GlyRs were seeded into 384-well plates. The culture medium was replaced by 20 µl control NaCl solution and cells were imaged once to record cellular fluorescence in unquenched state (grey dot at 0 µM glycine). This step was conducted to optimise auto-focussing and detection of fluorescent objects in recorded fluomicrographs. Cells were perfused with NaI solution containing indicated lycine concentrations, whereupon a series of 5 images was recorded every 1.5 s. The lowest and highest fluorescence intensity, recorded in the first and fourth/fifth image is colored blue and red, respectively, with the intensities in intermediate images colored grey. **B.** Data filtering. Images of fluorescent cells always contained a substantial amount of debris (e.g. dead cells, cells expressing YFP_I152L_ but not GlyRs or cells detaching during perfusion). To discriminate functionally relevant fluorescence signals from artefactual data, dose responses were filtered after automatic curve fitting. The histogram shows the average number (mean ± SD) of objects per image, averaged from 10 experiments, with 20 randomly selected wells per experiment before (black, cells & debris) and after filtering (striped, mainly cells) using either of the parameters EC_50_, slope (nH), R^2^ and ΔF as described in Methods. To achieve a maximal number of unbiased concentration responses all four parameters were combined for filtering (grey), resulting in approx. 50% of data points considered acceptable. **C.** Representation of normalized concentration-responses measured in a single well after filtering. In this example a total of 186 (grey) and 95 (black) cells were accepted and rejected, respectively. **D.** Scatter plot of EC_50_ and slope values derived from curves shown in panel C. ***P<0.0001 relative to untreated control, unpaired t-test.

To determine the optimal timing conditions for generating single cell-based concentration responses of GlyRs, we co-transfected HEK293 cells with YFP_I152L_ and α1 GlyR cDNA and seeded the cells at defined density of 2.5×10^5^ cells in each well of a 384-well plate. The next day, and approximately 1 h prior to imaging, the culture medium was completely removed and was replaced by 20 µl NaCl control solution. The 384-well plate was placed onto the motorized stage of our in house-built imaging system and cells were imaged once in control solution to record cellular YFP fluorescence in unquenched state (see grey dot at time zero in Fig. 2A). As YFP_I152L_ is almost insensitive to chloride, its fluorescence intensity is highest in NaCl solution allowing for optimal focussing into the optical layer of cells. Subsequently, cells were perfused with 10 µl NaI solution containing progressively increasing concentrations (0.1–300 µM) of glycine and were imaged after the addition of each new glycine concentration by a series of 5 images recorded at 1.5 s intervals. Fig. 2A shows a sample time-resolved single cell recording of fluorescence intensity changes resulting from exposure to the increasing glycine concentrations. Application of low glycine concentrations (0.1–3 µM) resulted in fluorescence quench, followed by decreasing fluorescence quench (grey dots) with a peak at 3 sec (3 µM) to 6 sec (0.1 µM) past receptor activation (red dots) indicating iodide efflux upon receptor activation possibly due to the newly-created outward anion electrochemical gradient enhancing iodide efflux through other transport pathways. Application of higher glycine concentrations (10–300 µM) resulted in fluorescence quench, followed by increasing fluorescence quench, indicating iodide influx via GlyRs into the cell. Red dots indicate time points allowing for largest dynamic range of the concentration response. We recorded fluorescence intensity changes immediately after solution delivery (Fig. 2A, blue dots) for two reasons. First, it avoids possible contamination of concentration-response relationships by other transport pathways, and second, it dramatically speeds up the rate of data acquisition compared to time points indicated by red dots. Fluorescence dose-response data were fitted automatically using a Levenberg-Marquardt algorithm provided by the LabView VI *nonlinear curve fit.vi*. Fitting-derived parameters are often biased by objects such as non-cellular fluorescent matter or by cells that either do not express GlyRs or are washed away during repetitive perfusion. We thus filtered the fitting-derived parameters using four quality criteria: goodness of fit (R^2^), dynamic range of fluorescence quench (ΔF), slope of the curve at half-maximal receptor activation (slope, nH) and half-maximal concentration of receptor activation (EC_50_). The settings for each parameter are listed above in Methods. Fig. 2B shows filtering statistics calculated from fluorescent objects recognized in 200 wells randomly selected from 10 independent experiments with 20 selected wells per experiment (n = 51469 objects, 5146.9±1823.8 objects/experiment, 257.3±91.2 objects/well). The columns display the mean number of objects (± SD) that were accepted per experiment after filtering by the parameters, EC_50_ (4731±1888; 90±7%), slope (4422±1828; 84±8%), R^2^ (4176±1999 78±13%) and ΔF (3611±876; 74±14%), respectively. When all four parameters were combined, approximately half of all recognized objects were rejected (2614±871 objects, 51±7%). Fig. 2C represents normalized rejected (black, n = 95) and accepted (grey, n = 186) concentration-response curves calculated from fluorescence signals measured in a randomly selected well (n = 281). The dot-plot in Fig. 2D shows the relation of EC_50_ and slope values. This representation was used throughout this study as it allowed clear visible discrimination of distinct functional populations. Allowing for rejected objects, the number of recorded concentration-responses per well is high. In average 131±41 individual concentration-responses were generated in one well adding up to ∼10,000 concentration-responses per 384-well plate with 60–80 wells per plate. To our knowledge, this level of data acquisition from single cells has not previously been achieved in a single experiment, i.e. from a 384-well plate.

### Quality Control and Assay Validation

**Figure 3:**
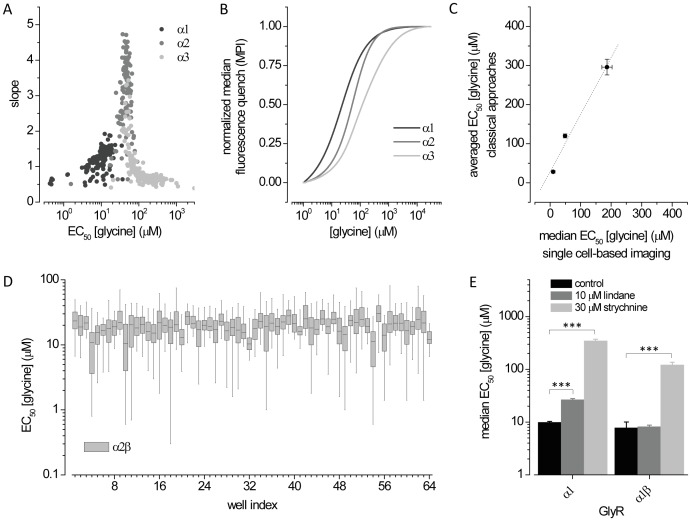
Quality control and proof-of-principle of the screening assay. **A.** Dot plot of glycine EC_50_ and slope values obtained from dose-response experiments with cells expressing α1, α2 and α3 GlyRs conducted in individual wells. **B.** Averaged and normalized concentration-responses from the experiment depicted in A. **C.** Comparison of EC_50_ values from the experiment shown in panels A and B to data from whole-cell patch-clamp electrophysiology (Islam and Lynch, 2011). **D.** Box-plot of glycine EC_50_ in α2β GlyRs measured in 64 wells of a 384-well plate demonstrating the stability and usability of the assay for operation in high-throughput mode. Boxes and whiskers display the 25–75% and 5–95% data ranges, respectively, of all accepted values in each well. **E.** Drug-effects of 10 µM lindane (grey) and 30 µM strychnine (light grey) on α1 and α1β GlyRs, validating our assay for identification and characterization of chemicals modulating GlyRs. ***P<0.0001 relative to untreated control, unpaired t-test.

To determine whether this assay could discriminate among different GlyR isoforms we transiently co-transfected HEK293 cells with halide-sensitive YFP_I152L_ and either α1, α2 or α3 GlyR cDNA. Fig. 3A displays single cell-derived EC_50_ and slope values for α1, α2 and α3 GlyRs recorded in individual wells as described above. Averaged EC_50_ values (in µM) are as follows: α1 (10.1±0.4, n = 147), α2 (49±2, n = 114) and α3 (186±17, n = 212). Slope values range between 0.47–1.92 (1.19±0.03), 0.51–4.73 (2.92±0.09) and 0.39–3.43 (0.91±0.03) for α1, α2 and α3 GlyRs, respectively. Averaged and normalized dose-responses from the experiment depicted in panel A are shown in Fig. 2B.

To evaluate how our method compares to whole cell patch-clamp electrophysiology, we correlated averaged EC_50_ values from the data shown in Fig. 3A with values previously measured in our lab (Fig. 3C). Regression analysis (dashed line) revealed excellent correlation with data from whole-cell patch-clamp electrophysiology (R^2^ = 0.99). Thus, our fluorescence-based method for physiological profiling of GlyR subtypes compares well with patch-clamp electrophysiology.

To assess inter-well reproducibility we recorded concentration-response relations from 64 wells of a 384-well plate containing cells co-expressing YFP_I152L_ and α2β GlyR heteromers. Fig. 3D shows a box-plot of glycine EC_50_ measured in single wells from a total of 6514 cells (102±35 cells/well). The mean single well-derived EC_50_ values as indicated by black line in displayed boxes vary between 10 and 30 µM glycine. The overall low inter-well variability indicates the assay is suited for high-throughput screening.

To test whether our assay is applicable to identification of drugs that specifically modulate homomeric or heteromeric GlyRs, we exposed cells co-expressing YFP_I152L_ and α1 or α1β receptors to strychnine or lindane while performing the glycine dose-response protocol as described above. Fig. 3E shows averaged glycine EC_50_ values of HEK293 cells expressing α1 or α1β GlyRs measured in single wells and exposed to control solution (α1∶9.8±0.5, n = 197; α1β: 7.8±2.3, n = 149) as well as to solution containing 10 µM lindane or 30 µM strychnine, respectively. As expected for a classical competitive antagonist that does not discriminate amongst GlyR subtypes [43], strychnine dramatically increased the glycine EC_50_ values of both receptors (α1∶346±26, n = 186; α1β 121±15, n = 205) (Fig. 3E). Lindane has previously been shown in electrophysiological studies to inhibit α1 but not α1β GlyRs [35]. Our fluorescence assay likewise demonstrates lindane antagonism of α1 but not α1β GlyRs (α1∶26.4±1.6, n = 156; α1β 8.1±0.6, n = 128) (Fig. 3E) thus validating our assay for identification and characterization of GlyR subtype-specific drugs.

Note that the strychnine sensitivity as determined here is lower than has previously been determined by electrophysiology or radioligand binding assays [10]. Because glycine accesses its receptor site quickly and strychnine access its site slowly, in co-application experiments as employed here, the YFP will be quenched (signalling full activation) before strychnine has had time to inhibit the receptors. Hence, super-saturating strychnine concentrations are required to enable it to bind rapidly enough to antagonise glycine activation.

### Case Study: Mode of Action of Lindane at Heteromeric α2β and α3βGlyRs

**Figure 4:**
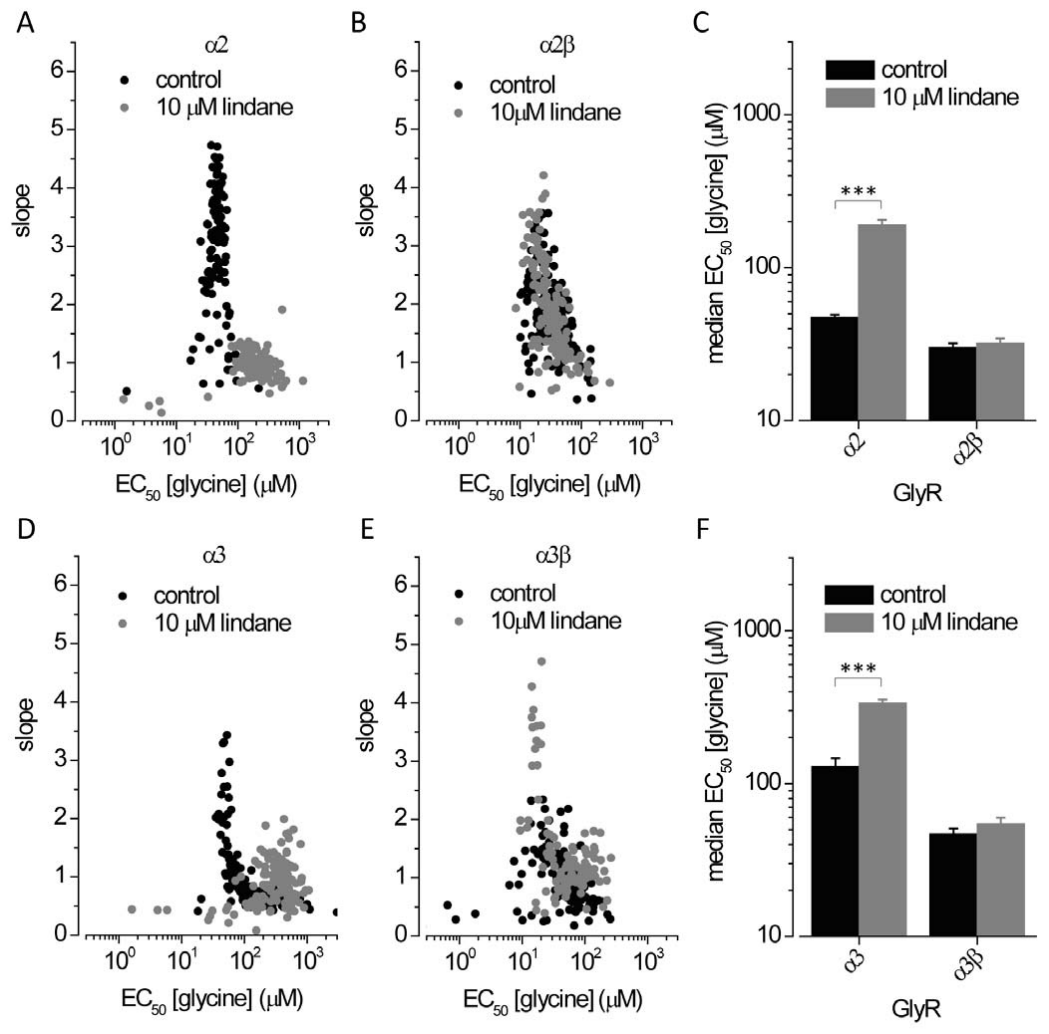
Effects of the lindane on α2, α3, α2β and α3β GlyRs as determined by fluorescence assay. **A, B, D, E.** Dot plots of glycine EC_50_ and slope values in absence (black) and presence (grey) of 10 µM lindane measured in single cells expressing α2 and α2β (A–B) and α3 and α3β (D–E) GlyR. **C, F.** Histogram of median glycine EC_50_ (±SD) calculated from data shown in panels A, B (C) and D,E (F). These results provide evidence for lindane as a pharmacological tool for identifying the presence of β subunits in α2β and α3β heteromeric GlyRs. ***P<0.0001 relative to untreated control, unpaired t-test.

It has recently been reported that the neurotoxic insecticide lindane (γ-hexachlorocyclohexane) selectively inhibits homomeric α1, α2 and α3 GlyRs but not heteromeric α1β channels [35] rendering the molecule an excellent pharmacological tool for identifying the presence of β subunits in functional GlyRs. However, its effects on α2β and α3β GlyRs has not been investigated. Cells expressing YFP_I152L_ and α2, α2β, α3 or α3β GlyRs were investigated in control NaI solution in the presence of NaI solution plus 10 µM lindane. Fig. 4 shows dot-plots of EC_50_ and slope values obtained from glycine dose-response recordings on α2 and α2β GlyRs (upper panels) and on α3 and α3β GlyRs (lower panels) in both the absence and presence of 10 µM lindane. Under control conditions average half-maximal α2 and α2β GlyR activation was observed at 47±2 (n = 114) and 30±2 (n = 166) µM glycine, respectively (Fig. 4C). Exposure to 10 µM lindane results in inhibition of homo but not heteromeric receptors (α2∶190±15, n = 106; α2β 32±2.5, n = 155). Similar results were obtained with cells expressing α3 homo and α3β heteromeric GlyRs (Fig. 4F). Under control conditions median half-maximal α3 and α3β GlyR activation was observed at 130±17 (n = 212) and 46.8±4.0 (n = 122) µM glycine, respectively. Exposure to 10 µM lindane results in inhibition of homo but not heteromeric receptors (α3∶337±18, n = 138; α3β 55±5, n = 106). These results confirm lindane as a pharmacological tool for identifying the presence of β subunits in functional GlyRs.

### Effect of Lindane at α2β and α3βGlyRs Assessed by Patch-clamp

**Figure 5:**
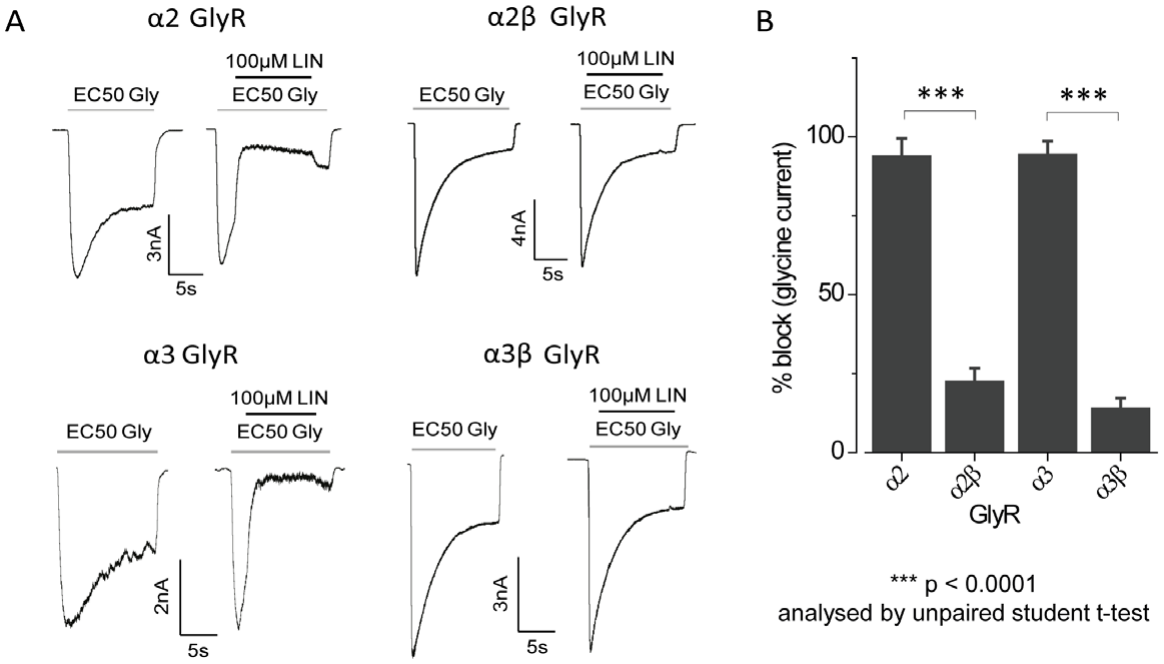
Effects of the lindane on α2, α3, α2β and α3β GlyRs as determined by patch-clamp. A. Examples of currents activated by EC_50_ glycine concentrations in HEK293 cells expressing the indicated GlyR subunits. Examples of the effects of 100 µM lindane on each GlyR isoform are also shown. B. Mean percentage (± SD) of EC_50_ glycine current blocked by 100 µM lindane. All results were averaged from 4–7 cells. ***p<0.0001 analysed by unpaired t-test.

The effects of lindane were also investigated on α2, α2β, α3 or α3β GlyRs using the whole-cell patch-clamp technique. As shown in the sample recordings in Fig. 5A, the effects of 100 µM lindane were investigated on currents activated by EC_50_ glycine in each of the four receptor types. Averaged results, presented in Fig. 5B, indicate lindane potently inhibited α2 and α3 GlyRs but had no significant effect on α2β and α3β GlyRs (% block, α2∶94±5, α2β: 23±4, α3∶96±4, α3β: 14±3). This validates the results obtained using our novel fluorescence-based assay.

### Functional Phenotyping of GlyRs in Heterogeneous Cell Populations

**Figure 6:**
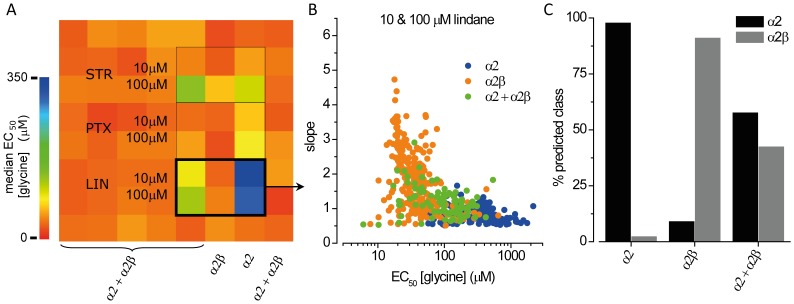
Clustering functional GlyR phenotypes in pure and heterogeneous cell populations. **A.** Color map of mean glycine EC_50_ (µM) in presence and absence of the drugs strychnine, picrotoxin and lindane, as indicated. Warm and cold colors represent low and high EC_50_ values indicating no effect and inhibition, respectively. The black rectangle highlights experiments shown in B and C. **B.** Dot plot of glycine EC_50_ and slope values measured in presence of 10 and 100 µM lindane in pooled pure (α2, blue; α2β, orange) and mixed (α2+α2βgreen, 1∶1 ratio) cell populations from the experiment in A. Values measured in pure populations were used for training a J48 decision tree classification algorithm. **C.** Functional phenotyping of lindane-exposed GlyRs in pure (α2, blue; α2β, orange) and mixed (α2+α2β green) cell populations. Cells were classified according to their descriptors with a training set of 402 cells distributed in 2 classes: α2 (black), α2β (grey). Bars represent the percentage of cells assigned to either of the two classes and reflect the initial 1∶1 mixing ratio (green).

For the sake of feasibility and throughput, conventional fluorescence-based ion channel-targeted drug screening is typically conducted in recombinant cell lines that express a single ion channel. The same is true for screening approaches targeting a specific ion channel subunit. Although multiplexing of various readout measures, e.g. different fluorescence indicators, is often applied this methodology is not applicable to parallel functional analysis of multiple subunits of the same receptor as the identical indicator is required for functional imaging of different subunits. Hence, when a large number of individual subunits are to be analysed, each isoform requires individual evaluation in serial mode that is time and cost intensive. To demonstrate the versatility of our assaying technique for parallel functional analysis of multiple GlyR subunits in heterogeneous cell cultures we applied the assay with pure and mixed recombinant cell lines and the GlyR inhibitors strychnine, picrotoxin and lindane. HEK293 cells were transiently co-transfected with halide-sensitive YFP_I152L_ as well as with α2 and α2β glycine receptor chloride channel cDNA and were seeded into 64 wells (8×8 wells) of a 384-well plate in the following pattern. A mixture of α2 and α2β GlyR expressing cells (1∶1 ratio) was seeded into columns 1–5 and 8. Columns 6 and 7 were supplemented with cells expressing α2 and α2β GlyRs, respectively. The agonist solutions were supplemented with strychnine, picrotoxin and lindane, each at 10 and 100 µM concentration and cells were used for functional screening as shown in Fig. 1. Fig. 6A shows a color map of median glycine EC_50_ (in µM) with cold and warm colors representing low and high values, respectively. The color coding indicates inhibition of α2 homomeric GlyRs by all drugs as well as α2β heteromeric GlyRs by strychnine but no inhibition of α2β heteromeric GlyRs by picrotoxin and lindane, validating previous data obtained using the assay as well as from the literature [43]. A dot-plot of glycine EC_50_ and slope values measured in presence of 10 and 100 µM lindane in pure (α2, blue; α2β, orange) and mixed (α2+α2β, green, 1∶1 ratio) cell populations from the experiment in A is shown in Fig. 6B. For clustering of functional GlyR phenotypes in pure and heterogeneous cultures we used the functional profiles of lindane-exposed cells expressing α2 and α2β GlyRs and trained a classifier using a set of 402 cells with 136 cells for class α2 and 267 cells for class α2β A 10-fold cross-validation on the training set revealed 97.8% (α2∶132 [97.8%)]; α2β: 3 [2.2%] cells) and 91% (α2∶24 [8.9%]; α2β: 243 [91.1%] cells) accuracy for α2 and α2β GlyR expressing cells, respectively. Cluster analysis using the model from cross-validation revealed 53 (57.6%) and 39 (42.3%) cells classified α2 and α2β, respectively. The bars in the histogram in Fig. 6C represent the percentage of cells assigned to either of the two classes and reflect the initial 1∶1 mixing ratio. The proportion of cells classified α2 in populations enclosing α2β GlyR is overall higher compared to the fraction of cells predicted α2β in cultures expressing α2 GlyR. These data suggest heterogeneous expression of α2 and α2β GlyRs in cell cultures transfected with α2 and β GlyR cDNA but were not further evaluated. Our results extend the applicability of the assay to functional phenotyping of GlyRs in heterogeneous cell cultures for identification of subtype-specific drugs and verify its suitability as a versatile platform for cell-based ion channel-targeted drug screening.

## Discussion

Large-scale analysis of ion channel function and systematic screening for subunit-specific drugs is hampered by the limitations of conventional assaying techniques. Despite available automated platforms, high-resolution patch-clamp electrophysiology is still limited in throughput compared to, e.g. fluorometric approaches that allow experimentation in high-throughput mode but that are low in resolution. For the sake of feasibility, throughput and cost reasons, large-scale functional analysis and ion channel-targeted drug screening are initially conducted in high-throughput mode using fluorometric approaches and identified candidates are subsequently analysed in more detail in secondary assays using either fluorescence-based or electrophysiological techniques. The initial benefit is often compromised by subsequent time-consuming and cost-intensive re-screens for confirmation of candidates and detailed characterization. To address this issue, we have developed an assay for functional profiling of ligand-gated ion channels, in particular GlyRs in single cells that is cost-effective, reliable and that is advantageous for several reasons. First, this assay is high-throughput compatible, thus allowing for efficient and thorough screening. Second, the approach makes use of little and cost-effective reagents, circumventing the need for expensive plastic ware and reagents, such as loadable fluorescence indicators. Third, this assay reveals a high number of single cell derived dose-responses and associated characteristics, such as EC_50_, slope (nH) and dynamic range of concentration response that allow large-scale analysis of ion channel function and systematic screening for subunit-specific drugs in pure and heterogeneous cell cultures.

We applied the assay with homomeric α1, α2 and α3 GlyRs and observed excellent correlation to EC_50_ values from patch-clamp electrophysiology, the gold standard in ion channel research, demonstrating the high level of accuracy of the assay and its suitability for assessing functional characteristics in single cells.

Despite the fact that a large proportion (∼50%) of all raw data generated using the assay were rejected there are still >100 individual dose-responses measured in a single experiment, i.e. well of a 384-well plate. At given conditions imaging of a single agonist concentration, including liquid-handling and image acquisition requires approx. 22 s per well. A complete imaging cycle for generating an eight to ten-point concentration-response, including auto-focus procedure and acquisition of control image adds up to a cumulative duration of 3.6–4.3 min per well allowing for screening analysis of 60–80 wells per 384-well plate. In this configuration up to 10,000 dose-responses are measured per 384-well plate resulting in ∼30,000 dose-responses per day with three 384-well plates per day. Measurement of a single dose response using the conventional fluorescence-based approach and our in house-built imaging system takes about 15 min suggesting an approx. 500-fold increased throughput for the novel assaying technique presumably outranging any other existing technique for assessing physiological properties of ion channels and highlighting the advantage of our approach over conventional fluorescence-based and electrophysiological methods.

We evaluated the quality of the assay to assess its suitability for high-throughput screening and observed robust operation and small inter-well variation implying its usability for high-throughput screening. Despite these results we were not able to use the full potential of the assay, e.g. for screening analysis of a complete 384-well plate due to limitations of our custom-designed hardware restricting the throughput to approx. 80 wells of a 384-well multititer plate. All experiments were conducted in extracellular solution at room temperature. Under these conditions and from our experience cells can be used in experiments for four to five hours limiting the time window for functional imaging. Implementation of infrastructure controlling environmental conditions, such as temperature and atmosphere allowing for long-term life-cell imaging would help to increase throughput and efficiency of the assay. Furthermore, iterative and sequential delivery of assay reagents using our in house-built imaging system is time-consuming and limits the applicability of the assay to non-desensitizing ion channels. Implementation of, e.g. microfluidic devices permitting rapid solution exchange [20], [44]–[46] or usage of caged agonists and flash-photolysis [47], [48] enabling concentration changes with fast kinetics could shorten cumulative imaging time, thus increasing throughput and extending the applicability of the assay to other types of ion channels.

We applied the assay in small-scale screening experiments with the drugs strychnine, picrotoxin and lindane and confirmed previously reported pharmacological profiles. Both picrotoxin and lindane have been reported to potently inhibit homomeric α2 GlyRs at the concentrations employed here [49], [35]. However, the color map in Fig. 6 A indicates overall less block of homomeric α2 GlyRs by picrotoxin compared to lindane. The two drugs produce inhibition by different mechanisms. Picrotoxin inhibitory potency is reduced as glycine concentration is increased [50]. In contrast, lindane inhibitory potency is not affected by glycine concentration [35]. In Fig. 6 we measured glycine EC_50_ values in the presence of fixed concentrations of each drug. As glycine should not easily be able to overcome non-competitive lindane inhibition, an extremely high glycine EC_50_ will be observed in the presence of lindane. In contrast, glycine should be more easily able to overcome picrotoxin inhibition, a more modest glycine EC_50_ increase is expected in the presence of picrotoxin. Our findings on the toxicity profiles of lindane at α2β and α3β GlyRs were validated by patch-clamp electrophysiology. These results demonstrate the accuracy of the assaying technique and its applicability to ion channel-targeted drug screening. Despite the fact that we applied the assay with drugs inhibiting GlyR it is equally applicable for identification of channel activators and potentiators.

To demonstrate the versatility of the method as a general platform for drug screening in heterogeneous cell cultures we applied our assay with lindane-exposed pure and mixed cell populations. The initial 1∶1 mixing ratio was reflected by cluster analysis. Remarkably, the proportion of cells classified α2 in populations including α2β GlyR was overall higher compared to the fraction of cells predicted α2β in cultures expressing α2 GlyR. These data suggest heterogeneous expression of α2 and α2β GlyRs in cell cultures transfected with α2 and β GlyR cDNA and validate observations from electrophysiology. When β containing GlyR are to be analysed by whole-cell patch-clamping is always necessary to confirm that the expressed receptors incorporated β subunits, e.g. by using a fluorescent protein cloned into the β subunit-encoding plasmid vector. While the assaying technique was initially applied for identification of subunit-specific drugs in mixed cell populations it provided additional information on heterogeneous GlyR expression, extending its applicability to functional analysis, that could be used e.g. for optimizing the expression of ion channels in recombinant systems or for creating comprehensive, large-scale and even time-resolved functional expression profiles of ion channels in cultures of primary or stem cells that are typically assessed in small-scale approaches using conventional methods. Single-endpoint or time-resolved expression profiles could be integrated with data from ‘omics’ approaches and help to gain a systems-level understanding of human physiology in maintenance and disease.

In the present example the heterogeneous culture was established from a known and small number of subpopulations and the applied analytical strategy using EC_50_ and slope values for prediction of classes has proven to be sufficient for clustering functional phenotypes. When more complex heterogeneous cell cultures, that consist of an unknown number of subpopulations, or mixed cell populations created from multiple cell lines are to be screened, more sophisticated analytical methods can be employed for interpretation of screening results. In this case additional single cell-derived functional characteristics, such as the dynamic range of the concentration response or even non-functional, e.g. morphological descriptors could be included for multiparametric analysis [51]–[53].

Our method uses a single indicator for functional characterisation allowing additional fluorescence or luminescence-based indicators to be implemented for multiplexing, e.g. for parallel analysis of further ion channels, to assess the activity of signalling pathways or for mapping cellular morphology to functional phenotypes. The combination of different assaying methods in the same experiment adds a level of efficiency by reducing sample supply, reducing cell culture and assay consumable requirements and eliminating redundant steps for sample preparation, plate replication, and assay execution.

In summary, our experimental and analytical method improves the classical fluorescence-based and electrophysiological assaying approaches for dose-response experimentation in terms of time, data content and cost. Based on our observations it is tempting to state that fluorometric functional imaging at the level of single cells enables accelerated characterisation of ion channels and ion channel-targeted drug screening compared to the classical approaches and eliminates the need for additional re-testing using independent assays. It is important to mention that the applicability of the described method depends on the ion channels to be evaluated and targeted, on the individual experimental setup, the available instrumental infrastructure and the biological question to be assessed. While this work focuses on functional profiling of GlyRs and GlyR-targeted drug screening, our method could also be adapted for other ion channels and strategies, such as RNAi or combined small molecule and RNAi screening, for single-endpoint or time-resolved functional expression analysis or for approaches using overexpression libraries. Altogether, this work contributes to furthering the applicability of cell-based high-throughput functional screening and provides a means for large-scale characterisation of ion channels in the context of cellular heterogeneity promoting a systems-level understanding of human physiology in homeostasis and disease.
